# Recovery of the spatially-variant deformations in dual-panel PET reconstructions using deep-learning

**DOI:** 10.1088/1361-6560/ad278e

**Published:** 2024-02-28

**Authors:** Juhi Raj, Maël Millardet, Srilalan Krishnamoorthy, Joel S Karp, Suleman Surti, Samuel Matej

**Affiliations:** 1 Department of Radiology, University of Pennsylvania, Philadelphia 19104, United States of America

**Keywords:** Breast-PET, dual-panel PET, deformation reduction, time-of-flight, deep-learning, limited-angle artifacts, spatially-variant PSF

## Abstract

Dual panel PET systems, such as Breast-PET (B-PET) scanner, exhibit strong asymmetric and anisotropic spatially-variant deformations in the reconstructed images due to the limited-angle data and strong depth of interaction effects for the oblique LORs inherent in such systems. In our previous work, we studied time-of-flight (TOF) effects and image-based spatially-variant PSF resolution models within dual-panel PET reconstruction to reduce these deformations. The application of PSF based models led to better and more uniform quantification of small lesions across the field of view (FOV). However, the ability of such a model to correct for PSF deformation is limited to small objects. On the other hand, large object deformations caused by the limited-angle reconstruction cannot be corrected with the PSF modeling alone. In this work, we investigate the ability of deep-learning (DL) networks to recover such strong spatially-variant image deformations using first simulated PSF deformations in image space of a generic dual panel PET system and then using simulated and acquired phantom reconstructions from dual panel B-PET system developed in our lab at University of Pennsylvania. For the studies using real B-PET data, the network was trained on the simulated synthetic data sets providing ground truth for objects resembling experimentally acquired phantoms on which the network deformation corrections were then tested. The synthetic and acquired limited-angle B-PET data were reconstructed using DIRECT-RAMLA reconstructions, which were then used as the network inputs. Our results demonstrate that DL approaches can significantly eliminate deformations of limited angle systems and improve their quantitative performance.

## 1. Introduction

Dual panel PET systems offer certain advantages, such as higher sensitivity because of the detectors being placed closer to the scanned object, easier access to the object (such as for biopsy and monitoring response to therapy), and a simpler, potentially cheaper, design (Surti and Karp 2008, Lee *et al* 2013, Krishnamoorthy *et al* 2020, 2021). On the other hand, limited angular coverage, truncated angular data, and strong depth of interaction effects due to the steepness of LORs given by the closeness of the detectors can lead to severe artifacts and point spread function (PSF) deformations, which are strongly asymmetric and spatially variant (Matej *et al* 2016). While TOF provides additional information, which reduces the limited angle artifacts, the problem is still quite challenging and cannot be eliminated even if a timing resolution of 200 ps or better is achieved (Gravel *et al* 2019, 2020).

In our previous works, we investigated effects of the TOF modeling (Gravel *et al* 2020) and application of the image-based resolution models within the statistical image reconstruction modeling the limited angle and Depth-of-Interaction (DOI) effects on the PSF deformations (Matej *et al* 2016, Gravel *et al* 2019). Although reconstructions with the optimized TOF kernel widths and with the spatially-variant PSF models led to better and more uniform quantification of small lesions across the FOV, the efficacy of the image-based resolution models is limited to small objects, such as point-sources and small lesions. Even though the reconstruction with PSF models showed more uniform contrast recovery of small lesions, their deformations could not be fully corrected with the PSF-derived image-based resolution modeling (IRM) alone. Furthermore, limited angle deformations of large objects cannot be recovered by this approach at all.

In (Li and Matej 2020) we proposed to use a DL based post-reconstruction approach to reduce or eliminate the limited-angle artifacts in the images reconstructed from a dual-panel system. The potential of the DL approach lies in the fact that the topology of neural networks, defining a hypothesis functional space, can be trained to provide a mapping from the data or deformed image to expected image with an ability to reduce the limited-angle artifacts (and rejecting any unrealistic null-space structures). In this study we continued with that work by further studying the ability of the DL based post-reconstruction approaches to capture and correct for the strong spatially variant deformations, as seen from the dual panel systems. It has been observed before that neural networks with variable resolution levels (of latent feature spaces), such as U-Net (Ronneberger *et al* 2015, Reader *et al* 2021), have the ability to capture the spatially variant image properties (Dai *et al* 2020, Shajkofci and Liebling 2020). However, to our best knowledge, nobody investigated so far, such a strong asymmetric (including shifts) and spatially variant deformation effects recovered by neural networks from those demonstrated in the dual panel systems.

**Figure 1.**
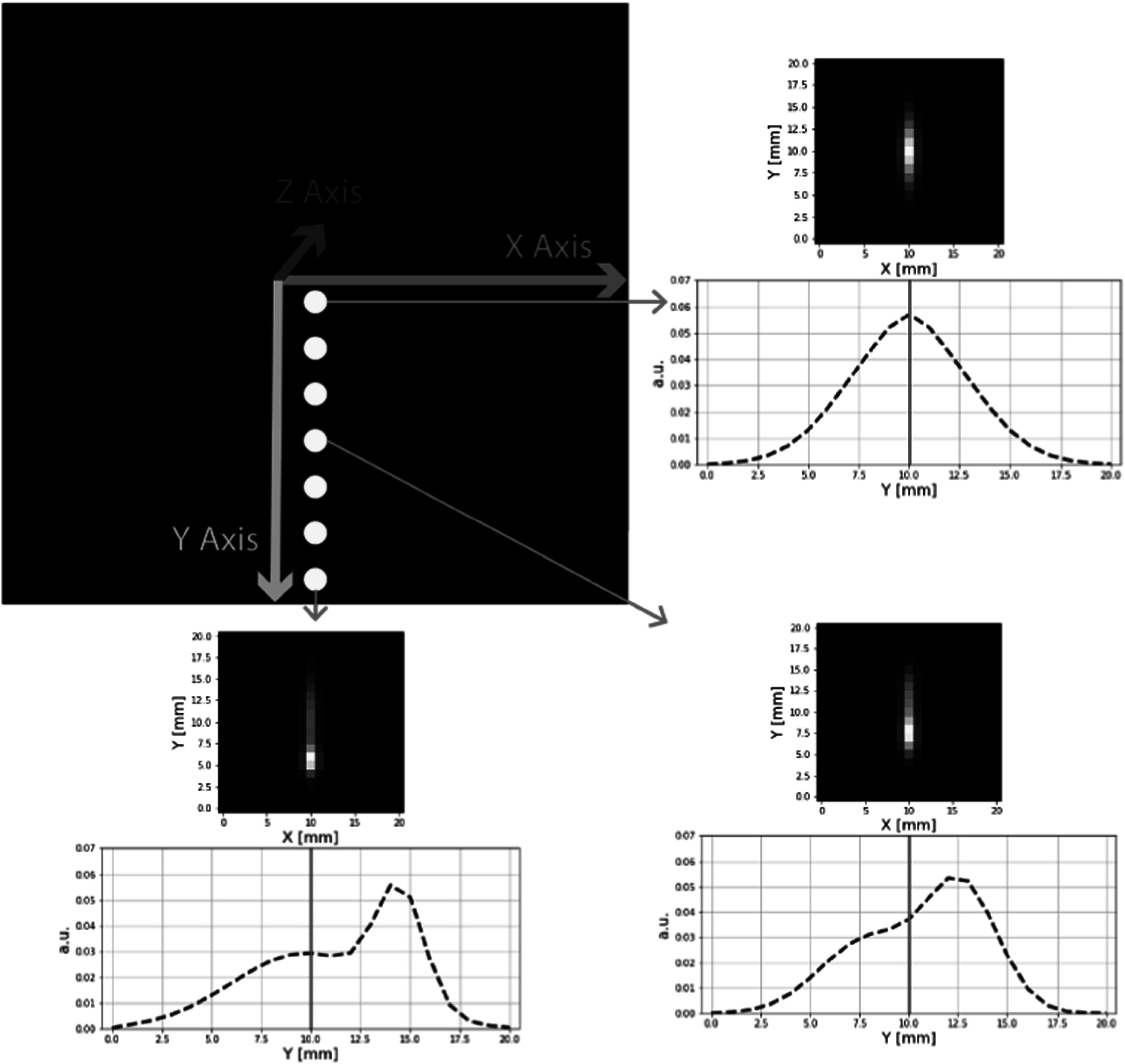
PSF resolution models applied in studies in section 2 using realistic PSF model based on the simulations of 3D PSF deformations in the reconstructed image space for point sources placed along the *y*-axis (with *y*-axis being normal to the detector panels). Illustrated profiles show PSF deformations in *y*-direction for 3 representative locations on *y*-axis. Deformations in x and z directions are similar to each other and are mostly symmetric. Each PSF kernel was parameterized using a Gaussian mixture model composed of the two 3D Gaussians with varying amplitudes, widths and shifts.

In the first part of this work, we focus on investigations of the ability of DL approaches to capture and recover such deformations, employing image-based PSF deformation models generated for a generic dual-panel PET system (Gravel *et al* 2019). In these investigations we simulated the image-based deformations (see figure 1) for range of the objects, from simple point-sources in air up to lesions of more realistic shapes in warm background and also incorporated the presence of noise to reflect real-world conditions. In the second part of the paper, we study performance of the post-reconstruction DL approaches to recover deformations of reconstructed images from the dual-panel B-PET system built in our lab at University of Pennsylvania (Krishnamoorthy *et al* 2020, 2021), including reconstructions and evaluations using synthetic realistic B-PET data, as well as real phantom data acquired on the B-PET system. Due to the limited amount of real data and lack of the true labels for the limited angle reconstructions, we have devised a solution to address the scarcity of real data and the absence of true labels for our limited-angle reconstructions. Our approach involves creating training datasets by generating reconstructions using synthetic data that model the experimental acquisitions. Network is trained on the synthetically generated training datasets and thereafter applied to both synthetic and real data reconstructions.

## 2. Ability of U-Net to capture the dual-panel PET deformations

### 2.1. Methods

As discussed and illustrated above, reconstructed images from dual-panel PET data exhibit strong spatially variant image deformations caused by the combination of limited angle data and depth of interaction effects. Deformations include limited-angle reconstruction artifacts, anisotropic PSF response with strong elongation in the direction normal to the detector panels, strong spatially variant asymmetry of PSFs, and their increasing shift in locations towards the detectors. These deformations cause spatially varying biases of the SUV values of small objects/lesions due to the PSF blurring effects, deformed object shapes, and their misplacements in the reconstructed images. To address these challenges, we first focused on a general question whether the neural network with variable resolution levels (U-Net) is able to capture such an extent of the spatially variant deformations as seen in the dual panel systems. To study this question we simulated PSF deformations of a generic dual-panel system with 120 degree acceptance angle of the detector panels and with deformation model based on a set of PSFs simulated for the grid of point-sources located in one quarter of the image space with 15 mm increments (figure 1).

The extent of the spatial variance of PSF deformations employed in these simulation was based on the image-based resolution models capturing the fitted PSF shapes (for grid of point-sources) in the reconstructed images from a dual-panel PET data (Matej *et al* 2016). PSF kernel for each point-source location was parameterized using a Gaussian mixture model composed of two weighted 3D Gaussians with varying widths and shifts (Gravel *et al* 2019). In the studies in this section focusing on the spatially-variant capabilities of the U-Net, the dual-panel PET reconstruction deformations were simulated by directly applying the spatially-variant PSF deformations to the label/test images. In the following section investigating U-Net performance on the practical B-PET scanner data, the PSF models were involved directly in the data simulations (forward projections) and the investigated networks were applied to the images reconstructed from such limited-angle data.

#### 2.1.1. Network architecture

**Figure 2.**
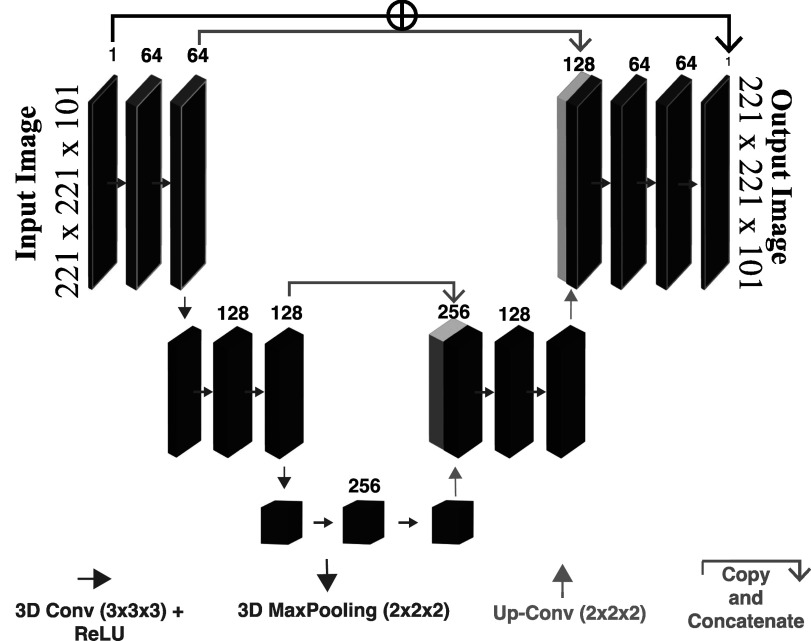
The 3D U-Net architecture adopted in this work. Each layer in the architecture is configured with 3 × 3 × 3 convolution kernels followed by a rectified linear unit (ReLU) as the activation function shown as red arrows. 3D MaxPooling function (pink arrow) was used in the down-sampling path. The Up-Transpose is depicted as yellow arrows in the up-sampling path. Skip connections are denoted as green arrows. The input and output image size is 221 × 221 × 101 with only 1 channel.

In our work, we adopted a 3D U-Net architecture, illustrated in figure 2. This network configuration is composed of three fundamental components: the down-sampling path, the bottleneck, and the up-sampling path. The down-sampling path is responsible for progressively reducing the spatial resolution while augmenting the number of convolution channels. With each down-sampling operation, the spatial resolution is halved, and the channel count is doubled.

The bottleneck captures and consolidates vital features and information from the input data. To restore the spatial resolution, the up-sampling path employs reverse operations, effectively ‘expanding’ the image, countering the effects of down-sampling. The network was designed to handle input images with a size of 221 × 221 × 101, representing images with 1 mm^3^ voxels. Each layer within the architecture consists of a 3 × 3 × 3 convolution layer, followed by a ReLU activation function, enabling the network to extract and process relevant features. The kernel stride of 2 was employed in both spatial down-sampling and up-sampling operations. Throughout the entirety of this work, presenting various deformation recovery tasks we present in this article, we consistently employed the same 3D U-Net architecture shown in figure 2.

#### 2.1.2. Network training

**Figure 3.**
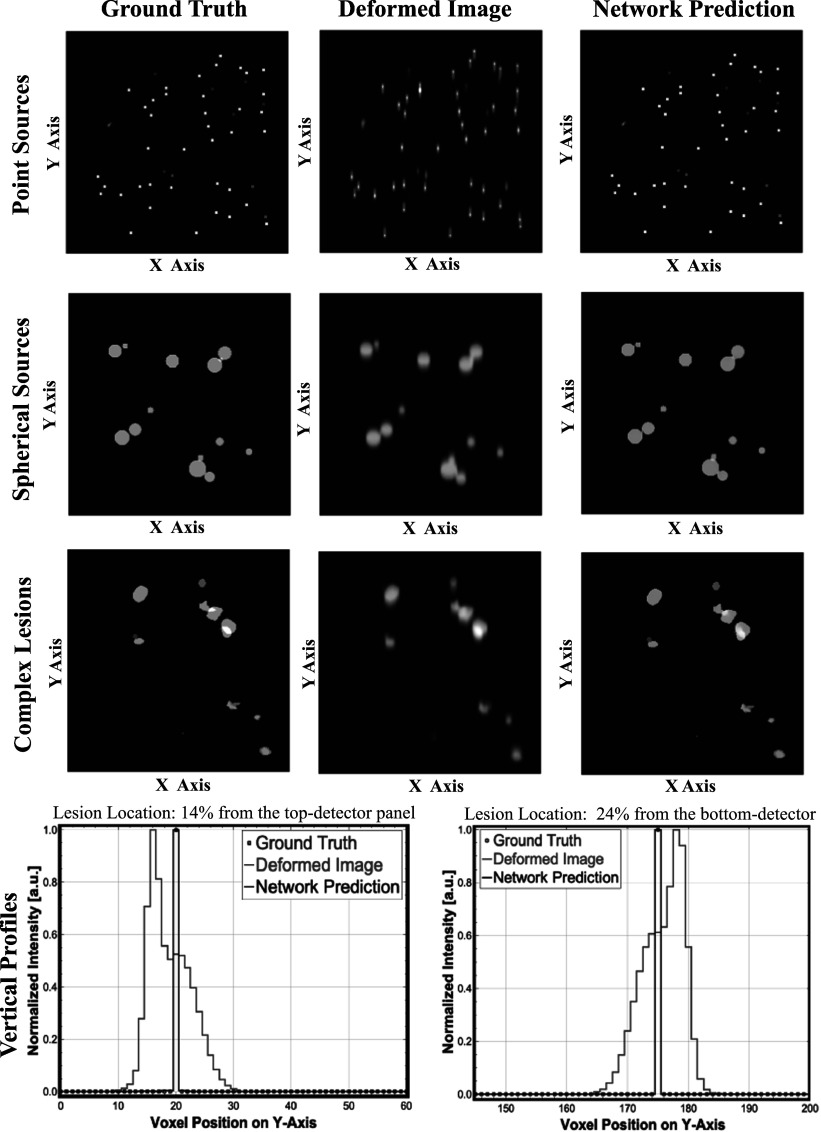
Top panel: Examples of studies using (training and testing) data sets of increasing complexity, using randomly generated point-sources (top), spherical lesions (middle), and complex lesions given by a combination of 3 randomized ellipsoids (bottom). Left column: Center slice of 3D ground truth images, Middle column: the corresponding deformed images, Right column: the network predicted outputs. Bottom panel: Vertical line profiles (along the *y*-axis) of small spherical lesions at different vertical positions (profiles are scaled, each curve by its maximum, to better illustrate their shapes).

**Figure 4.**
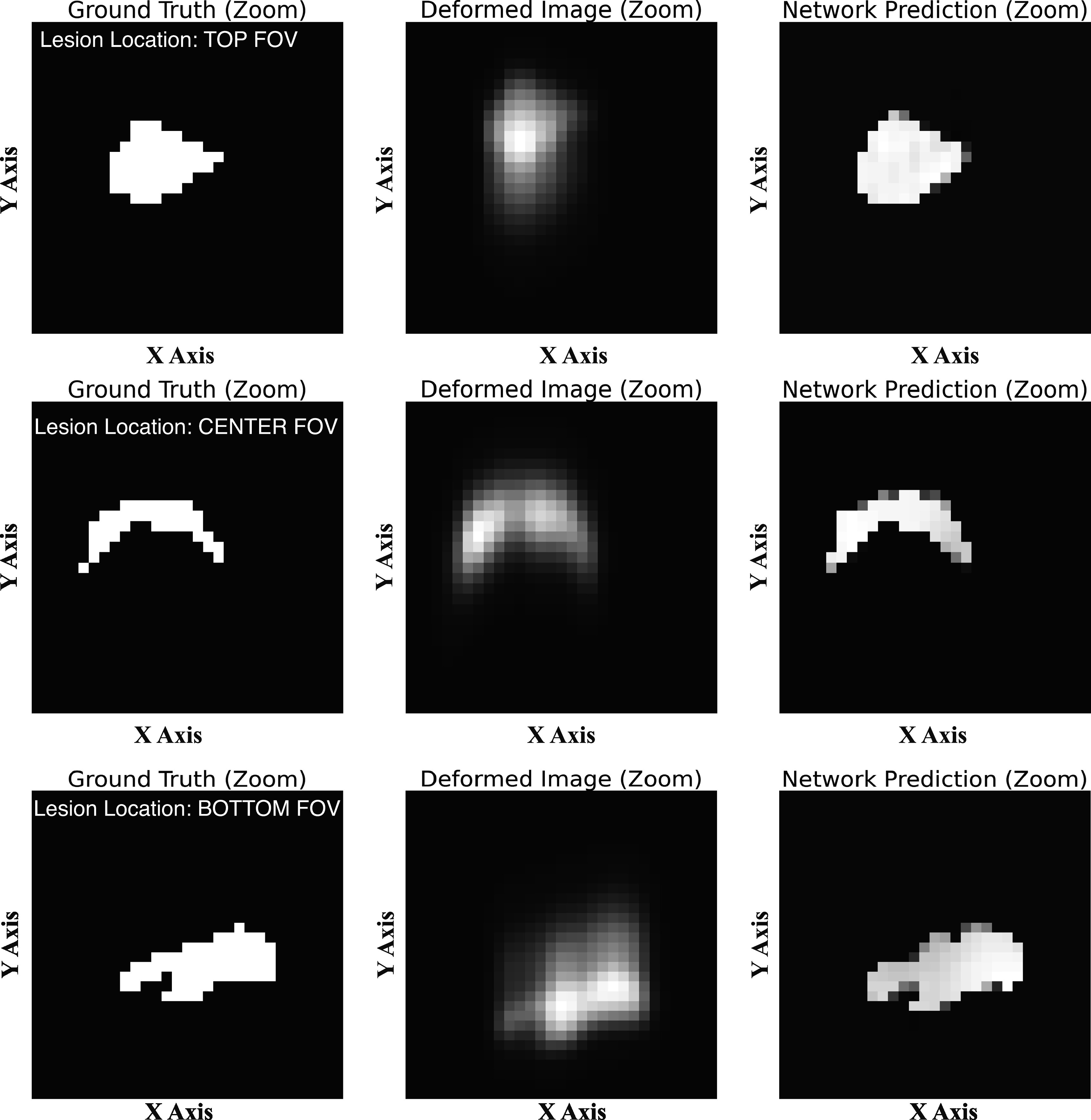
Examples of tests using realistic lesion shapes based on segmented lesions extracted from a conventional whole-body PET patient scan. Left column: central slices of the simulated lesion images (zoomed), Middle column: images with simulated PSF deformations (including spatially variant asymmetries and shifts), Right column: U-Net predicted images.

**Figure 5.**
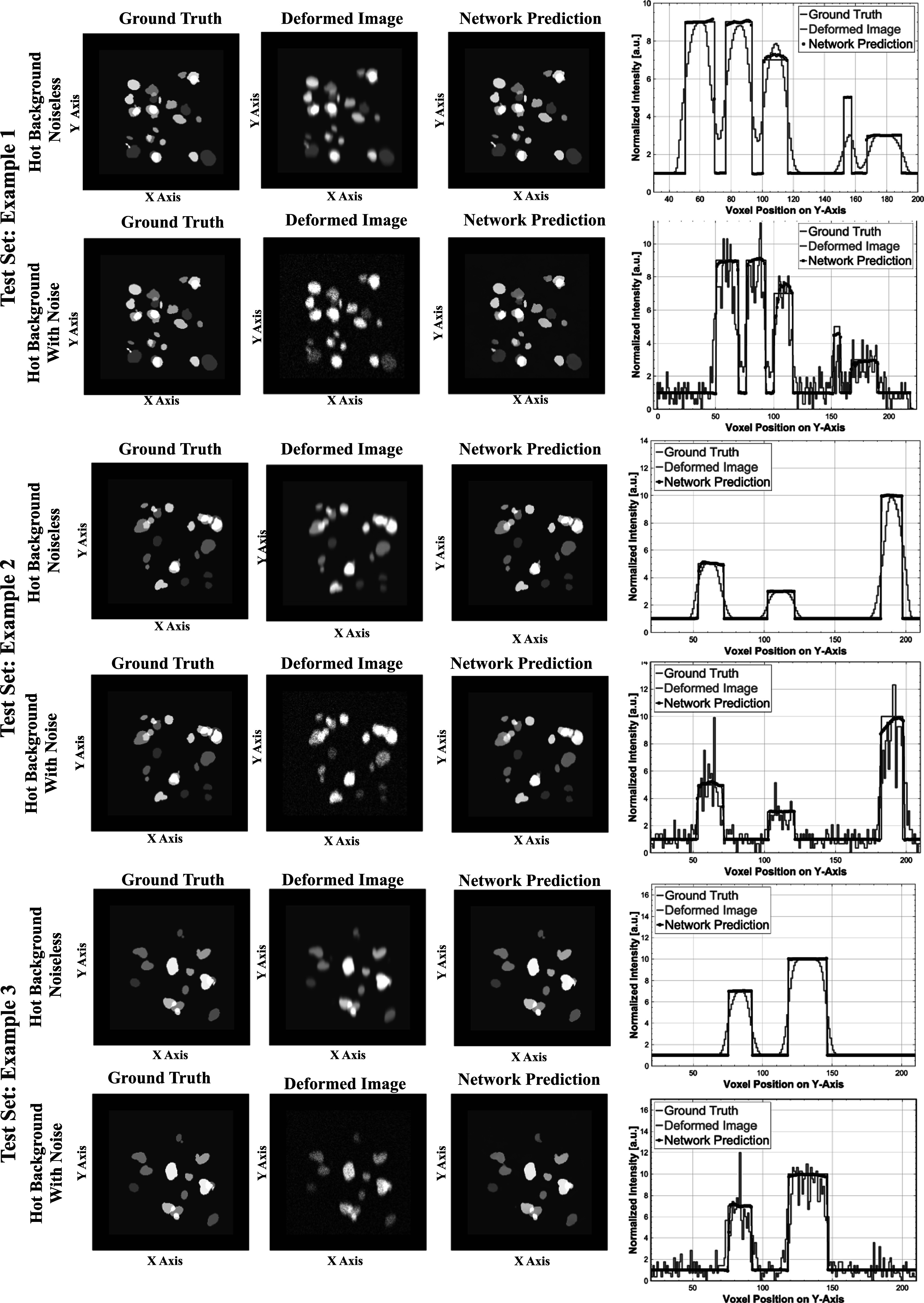
Three test examples of datasets with complex lesions in a warm background without (rows 1, 3, and 5) and with (rows 2, 4, and 6) image induced noise. Left column images: Center slices of 3D Ground truth images, Middle column images: corresponding deformed images, Right column images: the network predicted outputs. Plots on the right: Vertical line profile (along the *Y*-axis) through the three corresponding images.

For each of the following experiments, training and testing were executed separately for each case. Separate data sets were generated as illustrated in the figures 3–5, using images of point sources, spherical sources, and complex lesion-like structures (merging of three ellipsoids with random sizes and orientations) at random locations in the FOV with varying intensities. 50 training pairs and 5 test pairs were generated and used for each of the separate trainings and tests, respectively. The training was performed using the L1-loss function and Adam Optimizer with the learning rate 10^−4^ and a batch size of 1 for 5000 epochs.

### 2.2. Results

#### 2.2.1. Recovery of spatially-variant asymmetric PSF deformations in cold noiseless background

The top panel of figure 3 shows examples of the generated test phantoms, deformed images, and the 3D U-Net predicted images. The corresponding vertical profiles of reconstructed small lesions at two extreme locations (close to the detector panels) are shown at the bottom. We further tested the network trained on the complex (triple ellipsoid) lesions applying it to a set of the (PSF-deformed) realistic lesion shapes obtained by segmenting set of the lesions from a conventional whole-body PET patient scan (figure 4). Our studies using objects of variable complexity and realistic spatially-variant PSF deformations confirmed the ability of 3D U-Net network to substantially reduce the strongly asymmetric spatially-variant deformations and recover accurate lesion locations in dual-panel PET images.

#### 2.2.2. Recovery of complex lesions in warm background and noisy images

We have expanded our preliminary qualitative tests using objects in cold background presented in previous section to quantitative assessments using more realistic data sets with the lesion-like objects placed in a warm background of noiseless and noisy images (figure 5). Two separate data sets (50 training and 5 test data sets) were generated using complex lesion-like structures in a warm background with varying contrast-background ratio (2:1 to 10:1). The lesions-like objects of varying intensities were generated by merging three ellipsoids with random sizes and orientations with the total effective diameter of 6 mm to 30 mm and were placed at random locations in the FOV. In the first dataset (examples in the 1st, 3rd, and 5th rows in figure 5), the label/test images were just deformed with the spatially variant PSF models (as above) with no induced noise. In the second dataset (examples in the 2nd, 4th, and 6th rows in figure 5) generated image included also a realistic Poisson noise into the deformed images. The vertical profiles through the corresponding ground truth images, deformed images, and network predicted images for noiseless and noisy cases are shown to the right of the corresponding images.

The quantitative results of the test run evaluations are summarized in table 1 using the following metrics:

**Table 1.** Quantitative measures for evaluations involving test datasets with complex lesions in a warm background without and with image induced noise, illustrated in figure 5, assessing Average Absolute Lesion Bias (∣*Bias* %∣) over all lesions in the test images, average CRC over all lesions, and IR in the warm background.

Noiseless	∣*Bias* %∣	CRC	IR%
Deformed Image	17.92	0.79	0.00
Network Prediction	1.14	0.99	0.01

With noise			

Deformed Image	17.83	0.73	51.9
Network Prediction	5.24	0.94	0.41

**Average Absolute Lesion Bias** (∣*Bias* %∣): This metric quantifies average absolute bias over all lesions within the testing set, where the bias for a single *lesion*_*i*_ in an image *X* is calculated as:

\begin{eqnarray*}{{Bias}}_{i} \% =\displaystyle \frac{{\bar{X}}_{{{lesion}}_{i}}-{\bar{X}}_{{{true}}_{i}}}{{\bar{X}}_{{{true}}_{i}}}\times 100,\end{eqnarray*}

where ${\bar{X}}_{{{lesion}}_{i}}$ is the mean value within the volume of interest (VOI) of *lesion*_*i*_ and ${\bar{X}}_{{{true}}_{i}}$ is the mean value of the same lesion in the grand truth image.

**Average Contrast Recovery Coefficient (CRC):** The average CRC is calculated over all lesions within the testing set, where CRC for a single *lesion*_*i*_ in image *X* is calculated as:

\begin{eqnarray*}{{CRC}}_{i}=\displaystyle \frac{({\bar{X}}_{{{lesion}}_{i}}-{\bar{X}}_{{background}})}{{\bar{X}}_{{background}}}\div({{groundtruth}}_{{{contrast}}_{i}}),\end{eqnarray*}

where ${\bar{X}}_{{background}}$ is the mean value in the image warm background away from the deformed lesions, and ${{groundtruth}}_{{{contrast}}_{i}}$ is the ground truth contrast value for the *lesion*_*i*_.

**Image Roughness (IR):** This is an image noise measure assessed as the voxel-wise standard deviation (*σ*) in the background regions that are not affected by the blurring or deformations. It is calculated as:

\begin{eqnarray*}{IR} \% =\displaystyle \frac{{\sigma }_{{background}}}{{\bar{X}}_{{background}}}\times 100.\end{eqnarray*}

The qualitative results illustrated the figures 3–5 and quantitative results summarized in table 1 confirm the efficacy and robustness of the U-Net to recover the accurate shapes, locations, and uptake values of the deformed lesions in dual-panel images, when accurate representation of the deformations is known and utilized during the network training. This deformation representation can be known either through proper models, such as PSF deformation models used in the studies above, or through availability of training sets of (deformed) reconstructions from data generated for known ground truth images, as considered in the next section.

## 3. Deformation corrections of reconstructed dual-panel B-PET data

### 3.1. Methods

**Figure 6.**
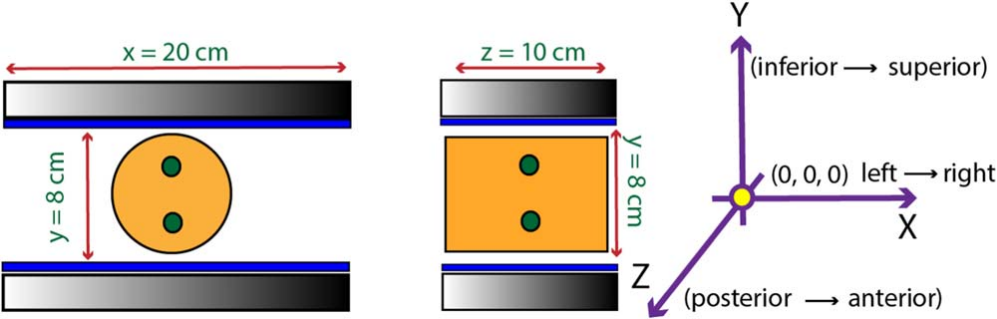
Transverse (left) and sagittal (middle) views of the dual-panel B-PET with a cylindrical phantom (yellow) with two lesions (green) placed in the FOV. The detector panels (grey) with the 0.5 cm compression panels (blue) are placed 9 cm apart. The axes on the right show the orientation of the detectors in physical space (with *y* pointing up towards the patients head and *z* in the direction away from the chest wall).

In the previous section, we studied the deformation recovery abilities of U-Net by modeling image deformations of a generic dual-panel PET scanner characterized by spatially-variant image-based PSF model. In this section, our primary focus is to assess the U-Net’s performance on reconstructed images obtained from a real-world dual-panel Breast-PET system. This evaluation encompasses the use of complete real data and takes into account the challenges posed by limited-angle reconstruction (including big object deformations). These studies involve data from the dedicated dual-panel B-PET system, developed in our lab at the University of Pennsylvania (Surti and Karp 2008, Krishnamoorthy *et al* 2020, 2021). The system is equipped with a detector configuration depicted in figure 6 comprising of two detector heads separated by 9 or 11 cm (with 0.5 cm compression pad on each side) giving the total transaxial angular coverage in the center of its FOV 132° or 122°, respectively (i.e. 73%–68% of the full angular coverage of 180°). Each detector head is composed of 4 × 2 blocks of PET detectors, with each block composed of 32 × 32 array of LYSO crystals measuring 1.5 × 1.5 × 15 mm^3^. In our study we utilized data from the initial prototype of the system with a 9 cm detector head separation, only half of the detector blocks activated in the *z*-direction, and with a time-of-flight (TOF) resolution of 500 ps (an update to the system has been recently completed providing 425 ps TOF resolution).

Both measured and synthetic (analytically simulated) data from the B-PET system underwent reconstruction using DIRECT-RAMLA, a reconstruction technique based on histo-image data partitioning, as described in prior work (Matej *et al* 2009, 2016, Gravel *et al* 2019). The data were histogrammed into histo-images with 1 × 1 × 1 mm^3^ voxels, 60 transverse views with 3° steps, and 15 axial views (tilts) with 3.3° steps (due to the use of only half of the blocks in the z directions, the total axial acceptance angle was limited to 50°). These angular sampling steps satisfied the angular sampling requirements (Daube-Witherspoon *et al* 2012) as given by the TOF and spatial resolutions of this system.

In the reconstruction, we used a relaxation parameter *λ* = 0.1, 10 iterations, no post-filtering, and no resolution modeling, i.e. the projection TOF/LOR kernel considered only the efficient LOR width (0.8 mm), size of the voxel (1 mm), and 500 ps TOF resolution (the relaxation parameter *λ* is a weighting factor applied to the iterative RAMLA updates of the image estimates, helping to control the rate versus stability at which the iterative reconstruction converges). The reconstruction process incorporated attenuation factors, normalization, and sensitivity correction, which were generated in the histo-image format (Matej *et al* 2009). It is worth noting that, for these relatively small-sized objects, scatter and randoms were not considered in the corrections. The normalization and sensitivity factors were determined experimentally and were consistently applied to both real and simulated data. Following the reconstructions, the U-Net was then applied as a post-reconstruction step to correct for deformations. For the network training, we used synthetic label images as the ground truth. In the evaluation phase, we compared the results obtained from the U-Net to those of DIRECT-RAMLA, which utilized an IRM as described in (Matej *et al* 2016, Gravel *et al* 2019). The reconstructions with IRM were post-filtered using a Han filter.

#### 3.1.1. Generation of training dataset for B-PET

The decision to use synthetic data in the training was primarily motivated by the two key factors: the unavailability of ground truth data and the limited access to real training data obtained from physical phantoms. Ground truth data, which serves as the absolute reference for model training and evaluation, is often challenging to obtain, particularly in the context of specialized PET systems like the dual-panel B-PET because of limited-angle artifacts and DOI effects. Real data often entails experimental measurements, which can be limited in quantity and may not cover the full range of possible scenarios or variations. To effectively train a deep learning model for a specialized system like the dual-panel B-PET, a large and diverse dataset is needed, enabling the model to learn from a wider range of scenarios and conditions, while using both synthetic and available real data for testing. The pipeline of generating synthetic training data pairs is described below. The trained network was tested on simulated test phantoms (phantoms not seen by the network during training; section 3.2.1) and experimentally measured phantoms (section 3.2.2).

The training dataset was composed of simulated cylindrical phantoms with a diverse range of characteristics. These phantoms were designed with varying diameters, spanning from 4 to 6 cm, and heights ranging from 4.5 to 5.5 cm. Within these phantoms, a range of 1–10 spherical lesion-like hot objects were randomly incorporated, with diameters ranging from 2 to 10 mm. Furthermore, the contrast ratios between the lesions and the surrounding tissue were deliberately varied, with ratios ranging from 6:1 to 10:1. To simulate real-world conditions, we introduced rotations of up to 6 degrees along both the *X*-axis and *Z*-axis. Importantly, we generated a total of 70 such phantoms, each placed at different positions within the FOV and displaying various orientations. These synthetically generated phantoms were employed as the ground truth labels for training the neural network.

**Figure 7.**
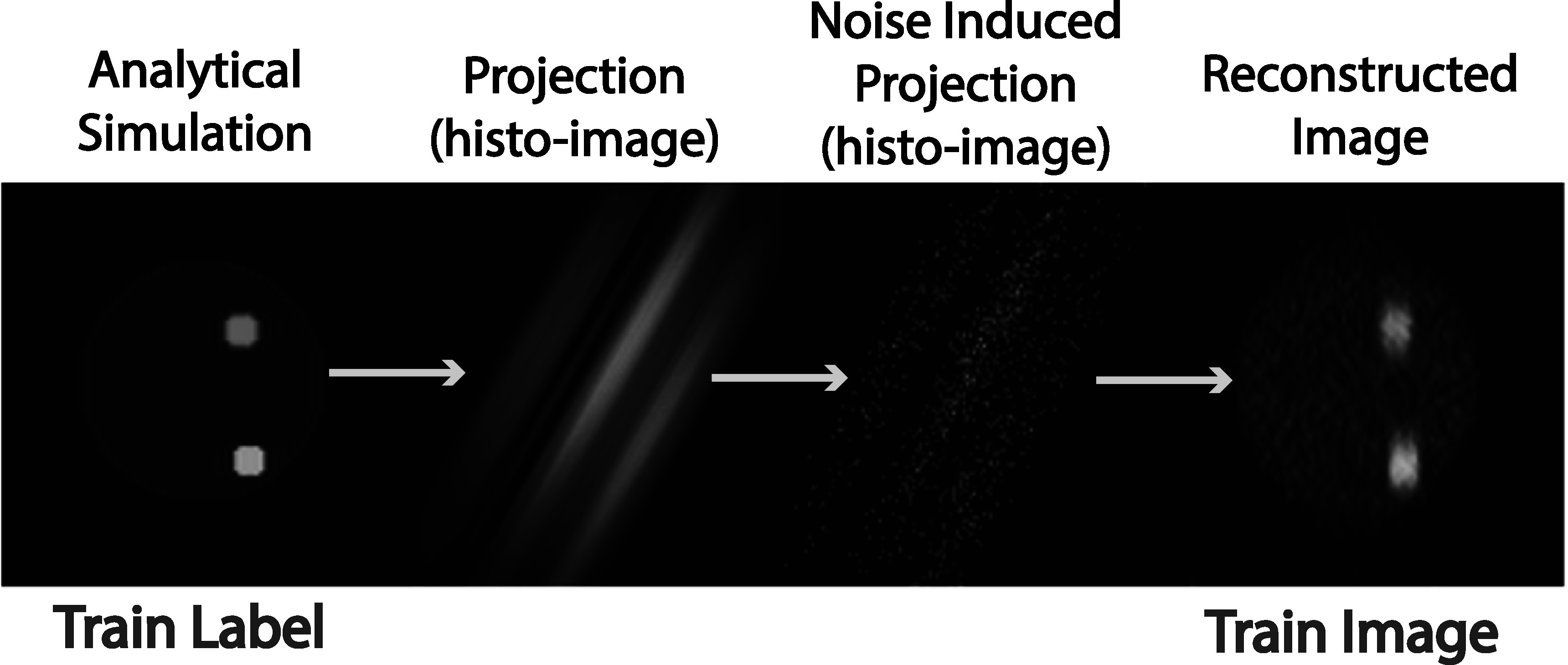
Pipeline of the training pair generation, starting with generated synthetic phantom (Train Label), followed by forward-projected histo-image, noise induced histo-image, and DIRECT-RAMLA reconstruction (Train Image).

The pipeline of generating a training pair is depicted in figure 7. On the left side of the image is an example of the analytically simulated phantom, which serves as the label for the training. This phantom image is then subjected to forward-projection, resulting in a set of histo-image views (the figure displays only one view out of multiple view histo-image). The forward-projection process includes considerations for B-PET resolution i.e. PSF models, attenuation factors, and the utilization of normalization factors obtained experimentally for the B-PET system. Subsequent to the forward-projection, the histo-images are subjected to Poisson noise (the noise levels in the generated histo-images in our studies were approximately matched to the noise levels in the histo-images deposited from the experimental phantom test data described in section 3.2.2), introducing a realistic level of noise into the simulated histo-image data. These noisy histo-images are then reconstructed using DIRECT-RAMLA algorithm, involving 10 iterations, without the implementation of IRM, and no post-smoothing. The image resulting from this reconstruction, shown on the right side of the figure, is used as the network input during the training process. The corresponding analytically simulated phantom on the left side is employed as the label, facilitating the training of the neural network. This training approach helps the network learn to correct deformations effectively by comparing the reconstructed images to the ground truth simulated phantoms.

#### 3.1.2. Network training

In our subsequent experiments, we applied the same 3D U-Net architecture, which was described in section 2.1.1 (figure 2). Our training dataset comprised 70 pairs of reconstructed images and their corresponding ground truth labels. In the training process, we employed the L1-loss function and utilized the Adam Optimizer with a learning rate of 10^−4^. Each training batch consisted of a single sample (batch size of 1), and the training process continued for a total of 10 000 epochs leading to 700k updates. This ensured that the neural network learned and adapted to the synthetic data, optimizing its performance for the given task. This trained network is then used in the all the following tests in this section.

### 3.2. Results

#### 3.2.1. Tests using simulated B-PET data

**Figure 8.**
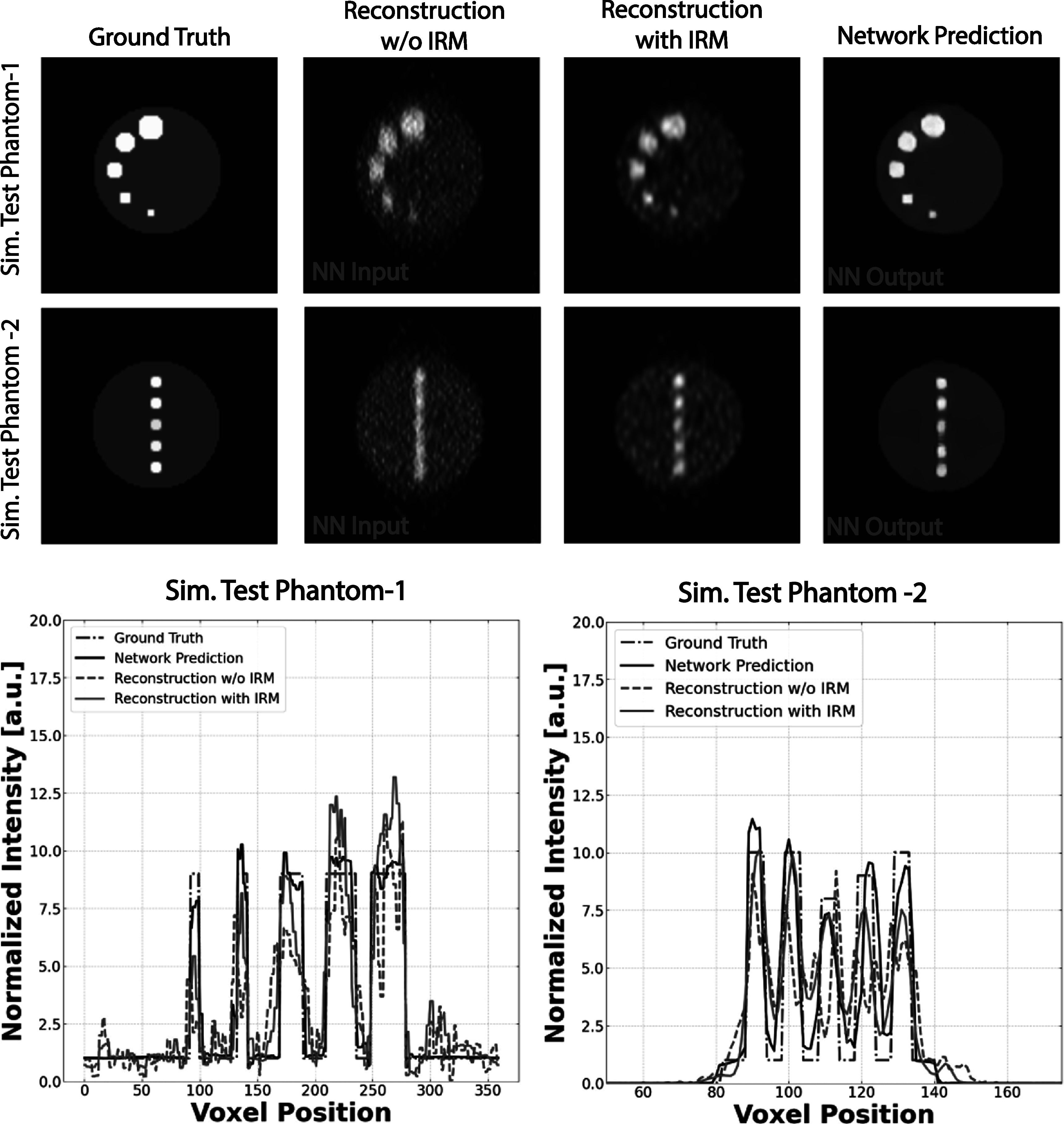
Test results for simulated B-PET data. Left column: phantom images, serving as the ground truths/reference images, second column: DIRECT-RAMLA reconstructions without IRM utilized as the network inputs, third column: comparative DIRECT-RAMLA reconstructions with IRM, fourth column: outputs produced by the 3D U-Net network. Bottom plots: profile lines through the phantom lesions in Test Phantom-1 (left) and Test Phantom-2 (right) images. Although the comparative reconstructions with IRM (green curves) provide considerably improved lesion contrasts compared to the non-IRM reconstructions (red curves), the improvement is clearly less consistent compared to the network output (black curves) and much more noisy (with a correlated lumpy noise).

Before moving forward with the validation of our trained neural network on experimentally measured phantoms, we conducted preliminary assessments using a collection of reconstructed simulation phantoms, which we have labeled as ‘Simulated Test Phantoms’. In figure 8, we display the central slices of two of these simulated test phantoms, identified as Simulated Test Phantom 1 and Simulated Test Phantom 2.

These simulated test phantoms are a valuable initial step, allowing us to evaluate the network’s performance under controlled conditions prior to its application to real-world data. Simulated Test Phantom 1, featured in the top row of figure 8, is a cylindrical phantom with a diameter of 6 cm and a height of 5.5 cm. Within this phantom, we introduced lesions of increasing size, ranging from 4 to 12 mm in diameter, each exhibiting a contrast-to-background ratio of 9:1. Simulated Test Phantom 2, displayed in the second row of figure 8, is another cylindrical phantom of the same dimensions but contains five lesions (closely placed in the *y*-direction with strong blurring), each with a 6 mm diameter and varying contrasts. The images presented in the respective columns offer a visual comparison of the following: the Ground Truth Image (analytically simulated phantom), the corresponding reconstructed image (obtained through DIRECT-RAMLA reconstruction and used as the network input), the comparative reconstruction with IRM (DIRECT-RAMLA with spatially variant IRM at 50 iterations), and the output generated by the 3D U-Net network. These visual comparisons provide an illustrative evaluation of the network’s performance on these simulated test phantoms.

The 3D U-Net demonstrates its capability to precisely recover not only the shapes of the deformed lesions but also the entire phantom itself. This capability is vividly illustrated in the vertical profile lines showcased in the lower panel of figure 8. The reconstruction with PSF models (i.e. reconstruction with IRM) demonstrated a more uniform performance as observed in comparison to the reconstruction without IRM, particularly in terms of representing small lesions. However, relying solely on PSF-based image resolution modeling was unable to completely correct the deformation exhibited by the phantom. In contrast, the application of deep learning yielded a notable enhancement in the quantitative accuracy of the images.

These improvements seen in figure 8 are further confirmed by the quantitative measures presented in table 2, evaluating the average absolute lesion bias (over all lesions in testing set), the average CRC, and the percentage of IR. When comparing the outcomes, it becomes evident that the neural network substantially enhances lesion accuracy, contrast recovery, and image roughness in contrast to conventional reconstruction techniques, thus validating its efficacy in addressing the deformations inherent in dual-panel PET systems. This is especially pertinent in the context of accurately recovering intricate structures and lesions within the phantom, emphasizing its potential for applications in medical imaging and improving diagnostic accuracy.

**Table 2.** Quantitative results for the simulated test B-PET data shown in figure 8, assessing Average Absolute Lesion Bias (∣*Bias* %∣) over all lesions in the test images, average CRC over all lesions, and IR in the warm background. These metrics confirm the network’s effectiveness in addressing the dual-panel PET deformations and improving the image quantitative quality on all reported measures compared the conventional approaches.

Sim. Test Phantom 1	Lesion ∣*Bias* %∣	CRC	IR%
Reconstructed Image	45.08	0.54	61.46
Reconstructed Image (with IRM)	30.06	0.79	51.97
Network Prediction	16.08	0.84	2.97


Sim. Test Phantom 2	Lesion ∣*Bias* %∣	CRC	IR%

Reconstructed Image	52.25	0.46	59.38
Reconstruction (with IRM)	41.61	0.61	50.14
Network Prediction	30.32	0.67	3.00

**Figure 9.**
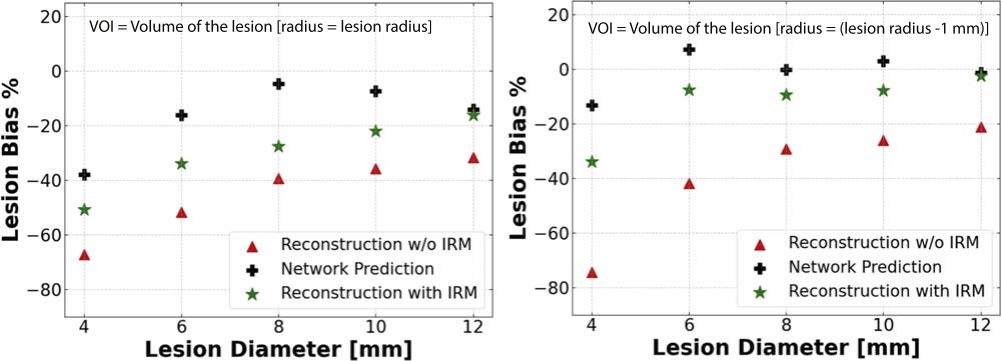
The lesion bias plotted as a function of the lesion diameter within the reconstructed image of Simulated Test Phantom 1. This analysis is conducted for both the images without and with IRM, as well as for the network’s output. The results are presented in two configurations, Left: the biases are assessed over the VOI defined by the full lesion mask. This indicates the biases calculated considering the entire lesion, including its boundaries. Right: The biases are assessed using a mask that has been reduced by one voxel (with a voxel size of 1 mm^3^) around the lesion’s perimeter. This approach eliminates the influence of voxels at the lesion boundary, focusing on the central region of the lesion.

In our analysis of Simulated Test Phantom 1, which features five lesions of varying sizes, we additionally conducted assessment of their individual biases and created plots to depict how these biases change in relation to the lesion diameter. Furthermore, our evaluation of the lesion biases extended to two distinct scenarios. First, we assessed biases over the full VOI, which is defined by the complete spherical lesion masks (as illustrated in figure 9-left). Second, we conducted bias evaluations using slightly reduced lesion masks, achieved by reducing the VOI radius by a single voxel (as seen in figure 9-right). This adjustment effectively eliminated the voxels located on the lesion boundary, where values transition between the lesion and the background. These two evaluation approaches were undertaken to gain a comprehensive understanding of the network’s capability to accurately estimate lesion bias within the simulated test phantom, while also considering the impact of the lesion boundaries on the obtained results.

**Figure 10.**
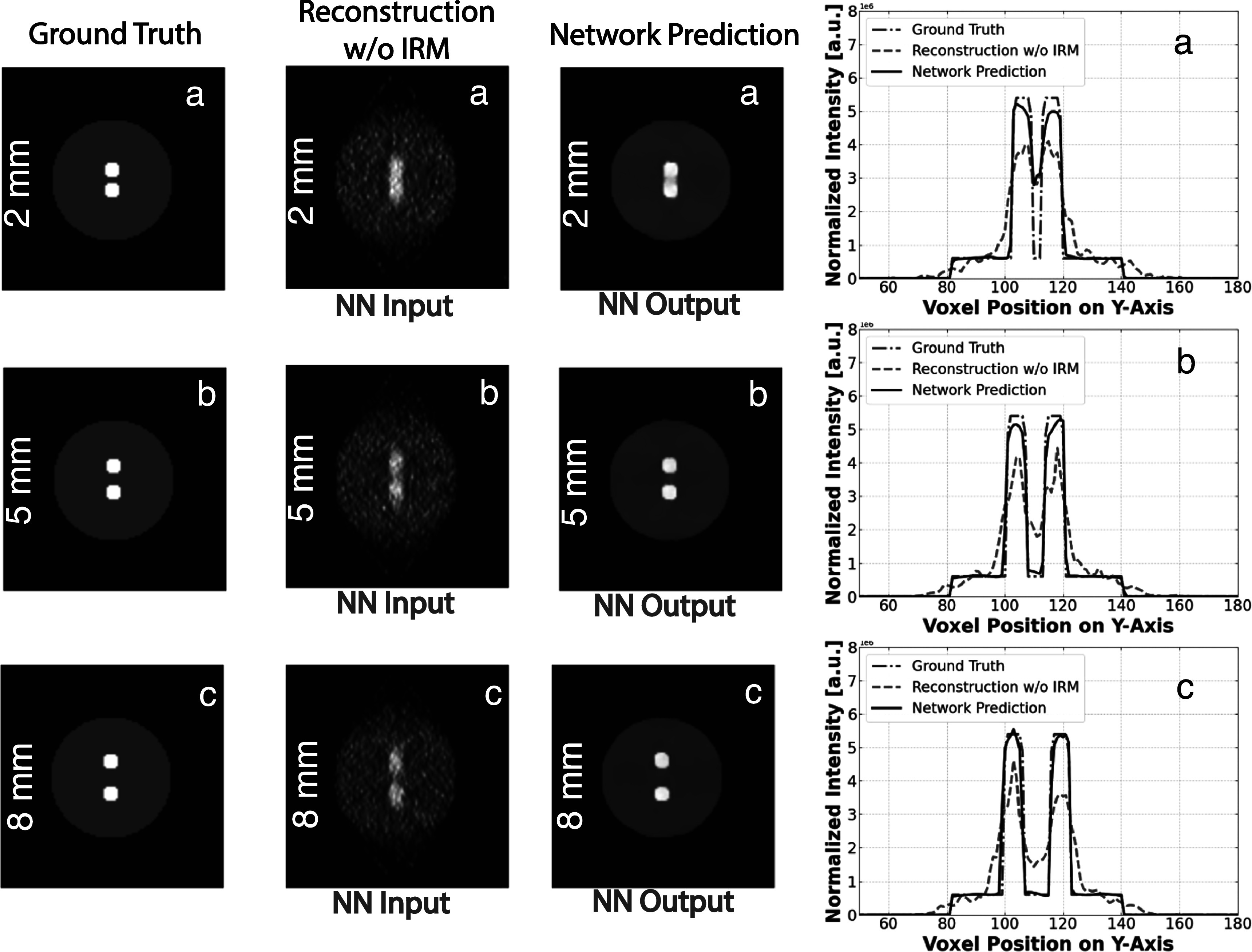
Lesion separability study: Illustrated images of three phantoms with 8 mm lesions positioned at different wall-to-wall distances (2 mm, 5 mm, 8 mm). Left images: ground truth images, middle images: reconstructed images, right images: network outputs. The corresponding 3 × 3 vertical profile lines through the corresponding images are shown on the right.

In our subsequent series of tests, we conducted a validation of our neural network using nine simulated cylindrical phantoms. Each of these phantoms had a diameter of 6 cm and a height of 5.5 cm. These cylindrical phantoms were designed to contain two lesions, each with an 8 mm diameter, positioned against a warm background. This arrangement resulted in a contrast ratio of 9:1, highlighting the differences between the lesions and the background. The unique aspect of these tests was the progressive placement of the two lesions at increasing distances from each other along the vertical *Y*-Axis. This configuration is illustrated in Simulated Test Phantoms 3a, 3b, and 3c in figure 10, where the distances between the walls of the lesions were set at 2 mm, 5 mm, and 8 mm, respectively. These tests were designed to assess the network’s performance in scenarios with lesions at different relative distances placed in the direction strongly effected by the dual-panel blurring.

**Figure 11.**
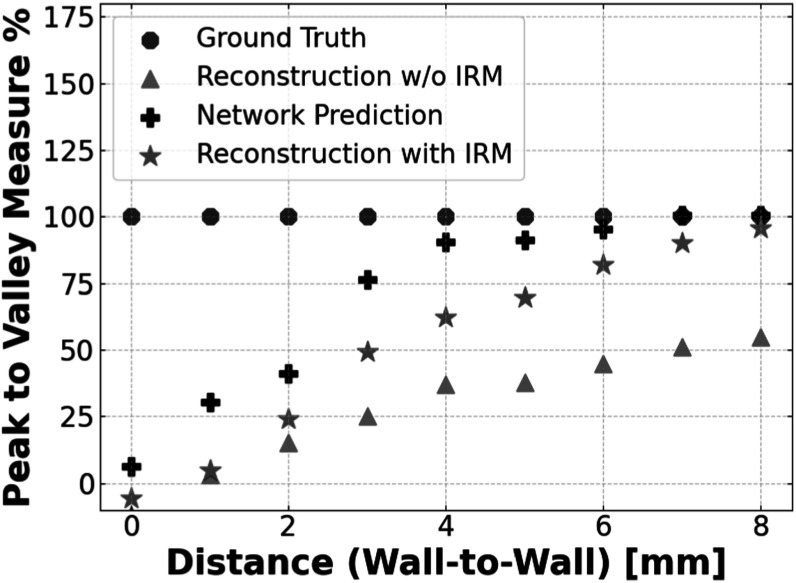
Lesion separability study: graphical representation of the peak-to-valley measure as a function of the wall-to-wall distance of two 8 mm lesions placed in the challenging *y*-direction with the trong blurring in a warm background.

The primary objective of this experiment was to assess the network’s ability to robustly and accurately recover the positions, shapes, and separability of lesions, especially when these lesions are positioned in close proximity to one another. To evaluate this, we measured the peak-to-valley measure (PVM) as a function of the separation between the lesions, which is represented in figure 11. The Peak-to-Valley ratio was determined by calculating the average profile formed by the 3 × 3 vertical lines passing through the centers of the spheres. The peak value (*P*) was taken as the average of the central values of the two lesions, while the valley value (*V*) was identified as the central value in-between the two lesions.

The peak-to-valley measure was then calculated as

\begin{eqnarray*}\mathrm{PVM}\ \% =\displaystyle \frac{P-V}{{\mathrm{groundtruth}}_{{PV}}}* 100,\end{eqnarray*}

where groundtruth_*PV*_ is the ground truth of the peak-to-valley measure. The results of this evaluation revealed that the network consistently and accurately recovers the lesion shapes and is able to separate close by features above 1–2 mm wall-to-wall separation. Notably, the network surpassed the performance of classical DIRECT-RAMLA iterative reconstruction methods in these scenarios. This showcases the network’s adaptability and its ability to generalize effectively, even when it has not encountered such specific instances during its training.

#### 3.2.2. Tests using experimental phantom data

**Figure 12.**
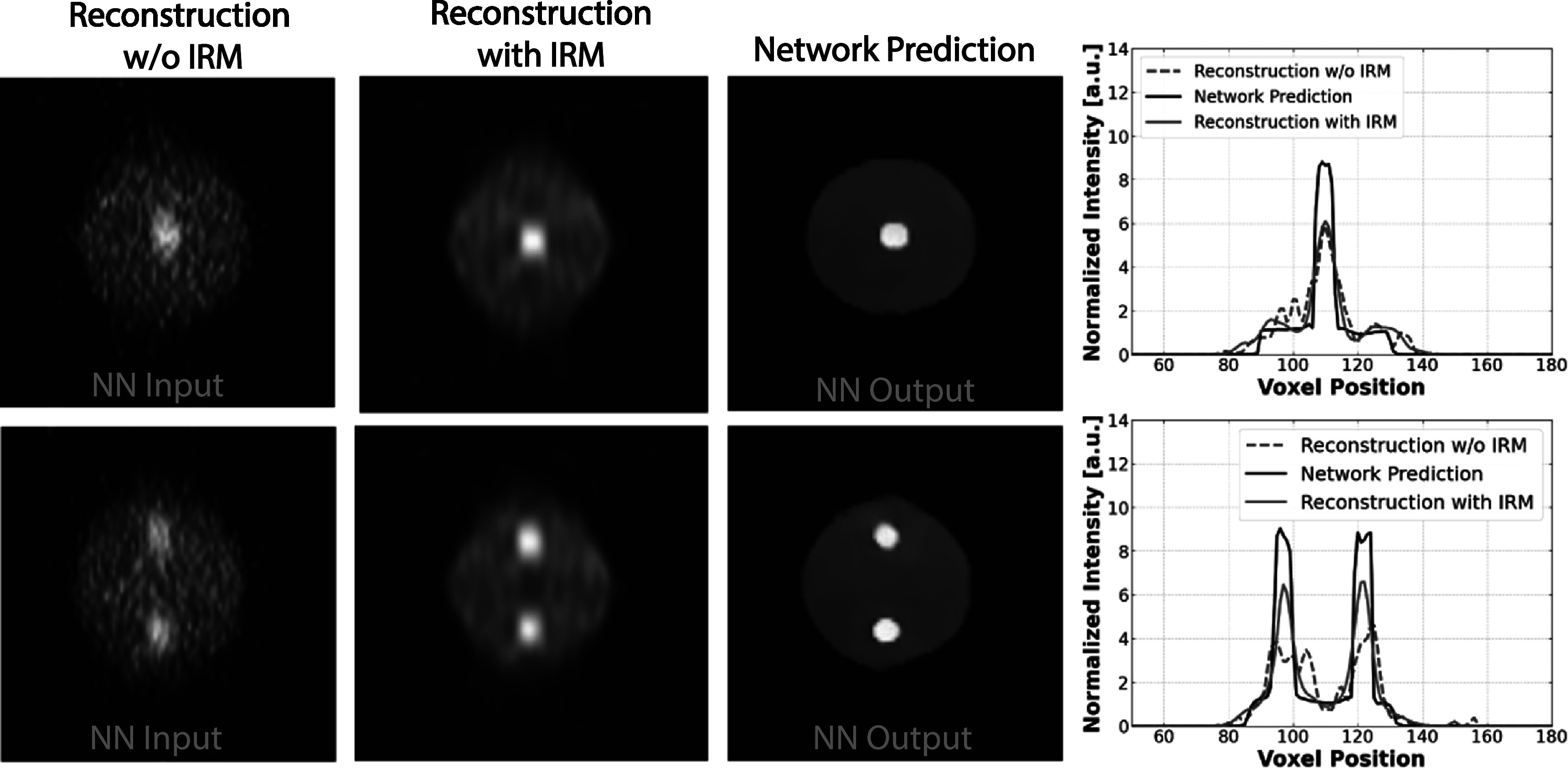
Tests using reconstructions of the experimental phantom data acquired on the initial prototype of the B-PET scanner. Left: DIRECT-RAMLA reconstructions without IRM (the network input), middle: comparative DIRECT-RAMLA reconstructions with IRM, right: 3D U-Net predicted images (with the network trained on the reconstructions from the simulated B-PET data). The corresponding average profiles of three central vertical lines across the images are shown at the right.

In the final phase of our study, we put our trained network to the test using experimental phantom data acquired on our B-PET scanner. It is important to note that the network had been trained on the simulated training dataset described in the previous section. The experimental phantoms used in this phase were cylindrical in shape, featuring a 4 cm diameter and a height of 5 cm. These phantoms were designed to contain 8 mm hot lesions within them. Specifically, one phantom included a single lesion placed in the center of the cylindrical phantom, while the second phantom featured two lesions of the same size positioned vertically along the phantoms Y-axis, as illustrated in figure 12. The B-PET scanner has been designed to acquire patient images with scan time of <5 min (Krishnamoorthy *et al* 2023). For the these phantom studies where we wanted to collect relatively high counts, we collected 4.3 Mcts with the single lesion phantom and 5.2 Mcts with the double lesion phantom, representing 15 min scan time for background activity concentration of 0.1 uCi/c.c. and contrast of 8:1 as corresponding to a typical clinical FDG scan.

For the image reconstruction, we used the DIRECT-RAMLA method without IRM for generating input images to be processed by the network. We also reconstructed the phantoms with IRM as a comparative reconstruction, providing a baseline for evaluating the network’s performance. This experiment aimed to assess how well the network performs on real-world experimental data while trained on the simulated synthetic data sets and compare its results with the conventional reconstruction approach with IRM. The reconstruction results and network outputs for the two phantoms and profiles through the central vertical lines of the phantoms are shown in figure 12, while the CRC and IR measures for the two phantoms are given in table 3 (CRC measure is based on the spherical VOI with diameter 6 mm centered on the lesion, CRC measure for the double lesion phantom is the average CRC over two lesions, and IR is calculated over a large uniform phantom region).

**Table 3.** The quantitative measures of the CRC and IR for the experimentally acquired phantoms displayed in figure 12.

Single lesion	CRC	IR %
Reconstructed image	0.48	38.8
Reconstructed image (with IRM)	0.65	15.9
Network prediction	0.85	2.9


Double Lesion	CRC	IR %

Reconstructed image	0.46	40.02
Reconstructed image (with IRM)	0.64	17.45
Network prediction	0.94	4.09

When comparing images and measures reconstructed with IRM and those processed through the neural network, several important observations emerge. First, the IRM reconstruction successfully reduces deformations in the small-lesion objects, leading to improved quantitative metrics like the contrast recovery coefficient. On the other hand, IRM reconstruction falls short in fully resolving the shape of the larger objects such as the cylindrical phantom itself. It also introduces/enhances noise correlations leading to their blobby appearance. In contrast, the network outputs reveal the U-Net’s ability to more faithfully capture the shape and characteristics of both the deformed lesions and the larger cylindrical phantoms. The significantly higher CRC of the network output indicates a substantial improvement in contrast recovery using the neural network and the lower IR% of the network output indicates excellent noise suppression both consistent with the visual observations. The results of network predicted images from experimentally acquired phantoms confirmed the feasibility of the deep learning model trained on synthetic data to be applied on real B-PET data.

## 4. Discussion

The purpose of this work was to investigate crucial questions related to DL approaches applied to images exhibiting strong spatial variant deformation such as those seen in dual-panel systems. This research had two main components: investigation of the ability of convolution neural networks (CNNs) to deal with such strong deformations, and development and test of a method to generate proper training data for such systems. The primary focus of this research was not to provide specific, practical network architectures, parameters, or loss functions tailored to a particular dual-panel application. Instead, it aimed to investigate the network’s capacity to handle data characterized by strong spatial variant deformations and to develop the necessary tools and methodologies for this purpose. By exploring the network’s adaptability and creating approaches for generating and evaluating training data in the context of such deformations, this work contributes to the foundation of knowledge and tools essential for addressing the challenges posed by dual-panel systems and similar applications.

While it is clear that fully connected networks can theoretically handle spatially variant behavior in imaging systems, their practical application to real 3D data is challenging, especially when compared to the suitability of CNNs. For CNNs, their ability to capture spatially dependent behavior through sequences of convolutions and network layers might not be immediately obvious. Nevertheless, it has been demonstrated that certain network architectures, like U-Nets, and the use of spatial encoding strategies can effectively capture and learn spatially variant models. However, it’s worth noting that, to the best of our knowledge, there was no prior research concerning the exploration of highly complex and spatially variant asymmetric and anisotropic image deformations, as observed in dual panel systems due to a combination of factors like limited viewing angles, depth of interaction, and data truncation effects. This motivated our investigations in this specific domain.

In our studies, we employed a relatively straightforward 3D U-Net with only three resolution layers, and it effectively captured the spatially dependent behavior of the dual-panel system for the specific synthetic object classes we studied. As we expand our works on the developed B-PET system and as more phantom and clinical data become available, we plan to undertake more extensive investigations and tests of the DL approaches using wider variety of realistic phantom and patient scans acquired with our new version of the B-PET system, with 425ps resolution. Our upcoming research will focus on assessing whether the shallow U-Net network we employed in this study suffices for more complex objects. We will also explore the use of deeper U-Net architectures and other network structures to further improve the correction of deformation effects. The shown results for the studies using experimental phantoms were designed to replicate breast PET images featuring one or few small high-contrast lesions in a fairly uniform warm background, resembling what would be encountered in actual breast FDG patient images. In order to apply this approach clinically (beyond breast imaging), we will need to further simulate the complexity of expected anatomy through analytical simulations. Subsequently, the neural network will need to be trained on the newly generated simulated data and tested on clinically acquired patient images. We will also investigate the generalizability of our training datasets to accommodate more diverse and challenging image classes, including patient scans with varying diseases or lesion types, and those scanned with different tracers. This will enable us to create more robust and versatile neural network models fine tuned for particular clinical applications.

In this work we successfully applied neural network to images reconstructed with DIRECT reconstruction without Image-based Resolution Modeling and let the network do the PSF corrections. We found DIRECT reconstruction without IRM to be more suitable for the practical use due to its faster convergence and lower computational costs compared to DIRECT with IRM. Additionally, the strong noise correlations introduced by IRM for dual panel-data with highly elongated PSFs and thus leading to blobby noise appearance in the reconstructed images might provide a challenge for the network in distinguishing small lesions from the blobby noise structures of comparable size. Nevertheless, other reconstruction approaches (as inputs to the DL pipeline), including the ones involving IRM, remain feasible candidates for the future investigations. Another promising approach proposed by us and which we plan to examine further, involves end-to-end deep-learning reconstruction directly from the B-PET data partitioned into the histo-image format, thereby eliminating the necessity for an intermediate reconstruction step.

One of the critical challenges in the context of neural network training is the generation of adequate and representative dataset, tailored to a specific application. Such a dataset should include pairs of ground truth images and input data images. The input data images may encompass various forms, such as noisy or low-count data, deformed images, or images with artifacts that require processing or correction. Constructing a high-quality dataset is pivotal in training neural networks effectively for a given task, as the network’s performance heavily relies on the diversity and quality of the training data it is exposed to. In tomographic applications, ground truth images typically consist of high-quality clinical reconstructions derived from data with favorable characteristics, such as high counts, full sampling, minimal motion, and low contamination. Neural networks are then trained to understand how to translate images obtained from lower-quality data, such as images with low counts or undersampling, into images resembling these high-quality references. The effectiveness of the network to improve the quality of the reconstructed images is inherently constrained by the quality of the label images (ground truth) within the training dataset. Essentially, the network can only learn to enhance images up to the resolution and quality present in the label images. It is challenging for the network to recover a better resolution or quality than what’s already available in these reference images.

However, a unique challenge arises in the context of dual panel systems. In clinical scenarios, it is often impossible to acquire high-quality images due to the limitations of the dual-panel tomographic system. For example, obtaining full angular data from a static dual-panel system like B-PET may not be possible. This absence of high-quality reference images in clinical data poses a distinctive challenge, making it difficult to apply the same image enhancement techniques as in cases with readily available high-quality labels. While it is feasible to obtain a collection of physical phantoms from which ideal or ground truth images can be generated, the practical constraints here involve both the limited quantity of these datasets and their inability to adequately represent the complexity required for effective neural network training. Here lies the importance of the results of this paper, confirming that it is possible to use properly simulated data for representative images retaining characteristics of given application (including data contaminations, resolution effects, limited-angle image deformations, and image characteristics) to train the network to be applied to real data. This approach enables the generation of data in any quantity and with the desired level of complexity, tailored to match the specific characteristics of a clinical application, which is essential for effectively training the network for that particular clinical context, encompassing factors like the tracer used and the clinical object under study. In our work, this was specifically tested using our labs dual-panel B-PET phantom data and simulations capturing the characteristics of these data. For any practical clinical application of this methodology beyond breast imaging it will be of crucial importance to carefully study and model the characteristics (including their variability) of the clinical objects and reconstructed images for the given clinical application to create representative simulated training data sets. This methodology can be extended to various tomographic systems, including whole-body PET, CT, or SPECT, as long as precise physical and mathematical models of the system and data are available, including system-specific norms and sensitivity corrections. This approach allows for the generation of high-quality ground truth data that is potentially more faithful and accurate when compared to clinical or specialized reconstructions obtained from actual acquired data.

Although the main motivation of our study was reduction of the deformations that result from reconstruction of limited angle data from a dual-panel scanner, a natural result of the DL approach is also a strong reduction (or elimination) of the image noise, as clearly demonstrated in our studies. This is even more so for the proposed approach when the network is trained on the synthetic data based on noiseless ground truth images. While improving the image quality and precision of SUV values, the noiseless images might look unnatural for clinicians used to look at the typical PET images, thus reducing their confidence in their diagnostic observations and assessment of SUV values. Noise in the images often provides certain information to experienced clinicians in judging the quality of data and reliability of their observations/SUV measurements. Using DL tools providing nearly noiseless images might require careful retraining of clinicians in interpretation of such images and perhaps development of supporting tools providing alternative information to clinicians (such as error bars on SUVs, or variance images) to improve their confidence in such DL generated images and to help them to gauge their interpretations. Another area of concern when introducing noise reduction approaches into clinical practice is to ensure that small structures, e.g. lesions, are not eliminated together with the noise. This will require careful specification of clinical tasks and realistic limits of the object sizes to be seen in the images of given modality and clinical application. These will then, in turn, dictate the class of objects to be included in the network training and the procedure for testing the networks to be sure that such objects are not eliminated during the network denoising process.

## 5. Conclusion

In this study, our primary focus was on assessing the effectiveness of CNNs in recovering spatially variant deformation effects present in dual-panel systems, which commonly result from limited angle artifacts and DOI effects. Our particular interest was in dedicated Breast-PET systems (B-PET). To tackle this challenge, we employed a 3D U-Net and utilized progressively complex datasets for both training and testing. Initially, we investigated the U-Net’s capacity to rectify these intricate spatially variant image deformations found in generic dual-panel systems and thereafter used the same network to train and test on simulated and experimental images from the dedicated B-PET system being developed at our University. For training the network on B-PET data, we created synthetic phantoms to serve as the ground truth (training labels) and used DIRECT-RAMLA reconstructions from the synthetically forward-projected data as the input for the network. Subsequently, we evaluated the trained network using simulated and experimentally acquired phantoms from the actual B-PET system.

Our experiments demonstrated that the deep learning approach, even when implemented with a relatively shallow 3D U-Net architecture, significantly reduced deformations in limited angle systems and notably improved the quantitative image quality within the context of B-PET. Importantly, this work exclusively explored the U-Net architecture for dual-panel deformation reduction. However, it highlights the broader potential of neural networks, especially when integrated with more advanced network structures, in addressing deformation and artifact challenges in dual-panel systems like B-PET or other modalities. This, in turn, could lead to enhanced image quality and diagnostic accuracy in such systems.

## Data Availability

The data cannot be made publicly available upon publication because they are not available in a format that is sufficiently accessible or reusable by other researchers. The data that support the findings of this study are available upon reasonable request from the authors.
